# Alcohol and morality: one alcoholic drink is enough to make people declare to harm others and behave impurely

**DOI:** 10.1007/s00213-023-06438-z

**Published:** 2023-08-09

**Authors:** Mariola Paruzel-Czachura, Katarzyna Pypno, Piotr Sorokowski

**Affiliations:** 1 Institute of Psychology, University of Silesia in Katowice, Grazynskiego 53, 40-126 Katowice, Poland; 2 Penn Center for Neuroaesthetics, University of Pennsylvania, Goddard Laboratories, 3710 Hamilton Walk, Philadelphia, PA 19104 USA; 3 Department of Neurology, Perelman School of Medicine, University of Pennsylvania, Philadelphia, PA USA; 4 Penn Brain Science Center, University of Pennsylvania, Philadelphia, PA USA; 5 Institute of Psychology, University of Wrocław, Wrocław, Poland

**Keywords:** Alcohol, Placebo, Moral foundations, Moral judgment, Sacredness

## Abstract

We aimed to understand if alcohol intoxication affects the willingness to violate moral foundations (care, fairness, authority, loyalty, and purity). We conducted a laboratory study (*N* = 387) with three randomized groups: alcohol intoxication, placebo, and control, measuring the sacralization of moral foundations via the Moral Foundations Sacredness Scale. The study showed intoxicated participants sacralized moral foundations of care and purity more often than participants from control and placebo groups. It means participants declared more willing to physically harm other people and animals and behave impurely, e.g., doing deviant sexual behaviors or selling their souls. No differences related to fairness, authority, and loyalty were found. Our study helps to understand the decision processes underlying immoral behaviors, including crimes. We showed that even one drink makes people change their judgments about what is right and wrong (in the cases of harmful and impure behaviors), and because this kind of judgment precedes immoral behaviors, our results may help explain why some people under the influence of alcohol break the rules by doing things which they would never do when sober.

**Supplementary Information:**

The online version contains supplementary material available at 10.1007/s00213-023-06438-z.

## Introduction

To function in the social world, people follow moral norms that guide their daily decisions and behaviors (Doris 2010). Moral foundations theory proposes that one way to categorize those values are five moral foundations, i.e., care, fairness, loyalty, authority, and purity (Graham et al. 2013, 2018; Graham and Haidt 2012; Haidt 2001, 2012). It was found that people differ in preferences for moral foundations, depending, for example, on their religious (Saroglou and Craninx 2020) or political preferences (Graham et al. 2009; Kivikangas et al. 2021). Different moral foundations may be sacred for some people and not for others (Tetlock 2003). “Sacred moral foundation” means that individuals insist their commitments to certain values are absolute and inviolable. This is why they would not, for any amount of money, violate the principles of that foundation (Graham and Haidt 2012). For instance, if someone sacralizes fairness, they would never take a bribe to produce any injustice. Because people are usually stable in their religious and political attitudes, it was assumed that preferences for moral foundations are also invariable (Haidt 2012).

The possibility of changing someone’s moral judgments, including moral foundations, even for a short time, was already discussed broadly in many fields (e.g., Berman and Small 2018; Bersoff 1999; Effron 2014; Kouchaki and Desai 2015; Monin and Jordan 2009; Moore 2015). However, the latest evidence suggests that moral judgments are surprisingly stable (see the review: Knobe 2021), and there is little chance that we can easily change someone’s most fundamental moral values. It makes sense as people start creating moral preferences from the earliest days of their lives (see the review: Bloom 2014). Thus, is there any way to at least periodically change someone’s preferences about moral foundations?

Since people make moral judgments based on emotion and reasoning (De Neys and Pennycook 2019), one possible way of changing moral judgments may be by manipulating an individual’s emotional and cognitive states, which can be achieved through the consumption of food or drink. However, can food or drink really change people’s behaviors, emotions, or moral judgments (Minhas et al. 2021; Schrieks et al. 2013)? Researchers have already provided a positive answer to this question. For example, consuming a high-carbohydrate/low-protein breakfast increased participants’ tendency to punish violations of social norms (Strang et al. 2017). This evolved mechanism may confer a survival advantage across evolution (Raison and Raichlen 2018).

Moreover, hunger changes moral disapproval of ethical violations (Danziger et al. 2011; Vicario et al. 2018). It has also been found that morality and appetite are linked, as well as morality and drinking. For example, drinking alcohol may be related to moral judgment (Duke and Bègue 2015). The potential influence of food and drink on our morality is no longer debatable, but the extent to which they can change us has not been precisely studied (Park and Schmid 2018). This article examines whether a small amount of alcohol can modify our willingness to violate moral norms.

We conducted a laboratory study among people under the influence of alcohol. Past studies consistently show that alcohol affects people’s thoughts, feelings, and behavior. Consumption of alcohol is known to influence a wide array of psychological processes, including emotional and cognitive ones (e.g., lower dispositional empathy or reduced executive cognitive functioning: Euser and Franken 2012; Giancola et al. 2010; Ray et al. 2012; Sayette et al. 2012; Thoma et al. 2013). Moreover, it changes the behavior so that one becomes more impulsive, violent, aggressive, and less sexually controlled, which can sometimes even lead to criminal violations (Exum 2006; Greenfield 1998; Heath and Hardy-Vallée 2015; Pernanen 1991; Roizen 1997). That is why we tested the hypothesis of the possibility of the sacralization of five moral foundations in a sample of alcohol-intoxicated participants.

### How people see the social world through moral foundations

Moral foundations theory has been the dominant theory of morality over the last decades (Graham et al. 2009, 2013, 2018; Haidt 2001). According to it, people differ in evaluating the importance of five moral foundations: care, fairness, loyalty, authority, and purity (Graham et al. 2018). The care foundation (the opposite of harm) relates to feeling empathy for the pain of others. Fairness (the opposite of cheating) concerns sensitivity to justice, rights, and equality. Loyalty (the opposite of betrayal) refers to the tendency to form coalitions and feel proud of being a group member. Authority (the opposite of subversion) relates to a preference for hierarchical social interactions and feeling respect for, or fear of, people in a higher social position. Finally, the purity (previously termed sanctity) foundation (the opposite of degradation) refers to a propensity to exhibit disgust in response to incorrect behavior and reflects individual differences in concerns for the sacredness of values (Koleva et al. 2012). Care and fairness are individualizing foundations because they are person-centered and focus on protecting individuals, whereas loyalty, authority, and purity are conceptualized as binding foundations because they focus on preserving one’s group as a whole (Graham et al. 2009, 2013, 2018). We already know that people differ in moral foundations, for example, because of religious reasons (Saroglou and Craninx 2020).

Studying moral foundations is not only relevant to understand how people think about right or wrong but also to predict their future behaviors. For instance, according to the theory of planned behavior (Ajzen 1985), individuals act following their attitudes, subjective norms, and perceived behavioral control (see the review: Kan and Fabrigar 2017). Indeed, many studies have confirmed that moral foundations are related to real-life behaviors. For example, low care has been found to be a predictor of violent behaviors (Vecina 2014), while higher binding moral foundations have been linked to group marijuana use and illegal phone use while driving (Silver and Silver 2021). However, it is worth noting that the relationship between beliefs and behaviors is complex, and even in the theory of planned behavior, it is not assumed that we can always predict someone’s behavior based solely on knowledge about their attitudes. Lastly, moral foundations are related to what people think and feel, as according to dual-system theory, moral judgments are the effects of both emotional and cognitive systems (De Neys and Pennycook 2019). That is why any emotional or cognitive manipulation may be useful in changing what people decide (or do) on moral issues.

### How alcohol changes people

First, alcohol affects mood and emotion. Alcohol intoxication is associated with increased emotional reactivity and selective attention toward emotional cues (Euser and Franken 2012; Giancola et al. 2010; Ray et al. 2012; Sayette et al. 2012). More specifically, drinking alcohol tends to improve individuals’ moods (Kuntsche and Cooper 2010), often stimulating euphoria in the immediate aftermath of consumption; alcohol consumption “creates the illusion of well-being” (Banaji and Steele 1989 p. 147) or makes people feel happier (Cohen et al. 1958; Corazzini et al. 2015) and may also be related to empathy (Francis et al. 2019). Positive emotions are more readily processed than negative ones (Carvajal et al. 2004), and people may make faster decisions when they are in a good mood. Moreover, individuals in a positive mood were more likely to engage in prosocial behavior and less likely to refrain from activities that have harmful consequences for others (Noval and Stahl 2017). Similar tendencies have been found in many areas. For instance, participants experiencing an optimistic mood exhibited greater dishonesty than those experiencing a pessimistic mood (Siniver and Yaniv 2019). Additionally, feelings of positivity increased decisions to push a man from the bridge in the famous footbridge dilemma, as authors suggested that it may have reduced the perceived negativity of potential moral violation (Valdesolo and Desteno 2006). Therefore, alcohol consumption may decrease the sacralization of the five moral foundations. In other words, it may increase self-reported violations of moral foundations as alcohol puts people in a better mood.

Second, cognitive reasoning is distorted in intoxicated people (Giancola et al. 2010), such that alcohol impairs cognitive control, reaction time, and memory (Weafer et al. 2016); therefore, sacralization of moral foundations could be biased as a result of decreased cognitive capacities. There are at least two ways that intoxication may play a role here, one regarding reduced cognitive focus (known as “alcohol myopia”) and the other regarding higher levels of disinhibition (Euser and Franken 2012; Heath and Hardy-Vallée 2015). Regarding the first way, alcohol myopia theory refers to the tendency of alcohol to increase a subject’s concentration upon immediate events and reduce awareness of distant events, which is why intoxicated people tend to focus on one central thing, idea, problem, or person (Steele and Josephs 1990). Thus, alcohol may lead intoxicated individuals to process information more simpler, with less nuance and balance than when sober, which may result in the lower sacralization of five moral foundations (people may choose not to think too deeply about the moral issues related to violating the moral foundations, and they may be more willing to openly declare their willingness to violate them).

Regarding the second way, one robust cognitive effect of intoxication is decreased inhibition (Giancola et al. 2010). Intoxicated individuals are more likely than non-intoxicated individuals to break the rules and overstep social norms. They tend to be more impulsive and less focused on the potential consequences of their unethical behaviors (Heath and Hardy-Vallée 2015). Therefore, when under the influence of alcohol and experiencing reduced inhibition, individuals may be more willing than usual to accept violations of moral foundations.

Third, alcohol affects behavior. For example, alcohol increases muscle relaxation (which usually makes intoxicated people more relaxed) and disinhibition (which can make people more confident and talkative) (Bodnár et al. 2021). Moreover, the social disinhibition associated with alcohol consumption can lead to impulsive, violent, and less sexually controlled behaviors (Exum 2006; Greenfield 1998; Heath and Hardy-Vallée 2015; Pernanen 1991; Roizen 1997). Suppose individuals rely on these behaviors when making moral judgments. In that case, it is reasonable to expect that people who consume alcohol may be more accepting of violations of moral foundations and more inclined to declare their willingness to violate them.

## The current research

In our research, we aimed to understand how alcohol may impact the sacralization of moral foundations. Following the research which shows that alcohol changes how people think, feel, and behave, we hypothesized that intoxicated participants would have lower levels of sacralization of five moral foundations than participants in control and placebo condition.

To test our hypothesis, we followed the study procedure of Francis and colleagues (Francis et al. 2019) and conducted a laboratory study with three groups: alcohol, placebo, and control. Instead of using the Moral Foundations Questionnaire (MFQ) (Graham et al. 2011), we employed the Moral Foundations Sacredness Scale (MFSS) (Graham and Haidt 2012). The MFSS measures explicitly the degree to which people sacralize the foundations, which aligns better with our hypothesis. Unlike the MFQ, which measures general attitudes, the MFSS focuses on direct questions about behaving in specific situations, making it more relevant to our study and hypothesis.

### Method

The study procedure was accepted by the Ethical Committee of the University of Silesia in Katowice. The study design was preregistered: https://osf.io/avf49. However, we deviated from it by adding the Moral Foundations Sacredness Scale. Data and code are available at: https://osf.io/bwg6m/?view_only=None. We report how we determined our sample size, all data exclusions, all manipulations, and all measures in the study.

#### Participants

This study was part of a larger preregistered study on moral decision-making (Paruzel-Czachura et al. 2023a); the sample size was determined by a power analysis seeking 80% power to detect an effect of *f* = 0.107 or *f* = 0.097 (depending on the type of measure), assuming a correlation of *r* = 0.30 between measures of moral decision making and nonsphericity correction of *ε* = 1, resulting in a target sample size of 300. Participants were recruited through media (e.g., university websites, Facebook, and newspapers). Only over 18 years, healthy individuals who did not report alcohol addiction or plans to get pregnant were eligible for participation. To verify these criteria, all individuals were asked to complete an online screening questionnaire before receiving an invitation to the lab study. Of the 1,079 volunteers who completed the screening survey, 387 met the criteria and were invited to the laboratory (198 women, 189 men; M_age_ = 25.7, SD_age_ = 7.4; age range = 18–52 years). Informed consent was obtained from all those participants. Participants were also asked not to drink alcohol for 24 h, not to take any medication (e.g., painkillers) for 10 h, and not to eat for at least 3 h before the study. Following the preregistered exclusion criteria, data from 58 participants could not be analyzed because they did not pass one or more of the attention checks in the survey. The final sample included 329 participants aged 18 to 52 years (*M* = 25.1, *SD* = 6.2). Of the 329 participants in the final sample, 106 participants were in the alcohol condition (53 women, 53 men), 114 in the placebo control condition (57 women, 57 men), and 109 in the no-alcohol control condition (53 women, 56 men). Following the preregistered stopping rule, we ended the data collection on the day we reached our target sample of 300 participants but included the data from all participants who came to the lab on the same day. This led to 29 participants over our target sample of 300 participants. After this day, all future appointments were canceled. Because our sample size differed a little from the one preregister, we additionally conducted post hoc sensitivity power analysis (*F* tests, ANOVA, repeated measures, within-between interaction) for *N* = 329 participants, 5 moral foundations, 80% of power, alpha level = 0.05, 3 groups, and the effect size was on a small, satisfactory level of *f* = 0.0678.

#### Measures

##### The moral foundations sacredness scale

This 20-item scale measures how people sacralize each of the five moral foundations, i.e., care, fairness, loyalty, authority, and purity (Graham and Haidt 2012). We asked participants to indicate the amount of money for which they would be able to do the described actions that violate the five foundations in various ways (e.g., “Stick a pin into the palm of a child you don’t know” for care; “Say no to a friend’s request to help him move into a new apartment, after he helped you move the month before” for fairness; “Say something bad about your nation (which you don’t believe to be true) while calling in, anonymously, to a talk-radio show in a foreign nation” for loyalty; “Curse your parents, to their face. (You can apologize and explain one year later)” for authority; and “Attend a performance art piece in which all participants (including them) have to act like animals for 30 min, including crawling around naked and urinating on stage” for purity. The instruction was: “Try to imagine actually doing the following things, and indicate how much money someone would have to pay you, (anonymously and secretly) to be willing to do each thing. For each action, assume that nothing bad would happen to you afterwards. Also assume that you cannot use the money to make up for your action”. The scale varied from 1 (*I’d do it for free*) to 6 (*I’d do it for 1 million dollars*). It was also possible to decline action for any money (7), which would be the maximum degree of sacralization of the specific moral foundation. The results for five moral foundations were created by averaging the subscale items on the full 7-point scale. We used the Polish version of the scale (Paruzel-Czachura et al. 2023b). The Cronbach alphas for the care (*α* = 0.68), fairness (*α* = 0.65), loyalty (*α* = 0.59), authority (*α* = 0.66), purity (*α* = 0.53), as well as for the full version of the scale (*α* = 0.86), were found to be satisfactory.

#### Procedure

The experiment was performed in accordance with the relevant guidelines and regulations of the Ethical Committee of the University of Silesia in Katowice. Three research assistants were actively involved during the study. The first assistant measured the participants’ weight and BAC (to check if everyone who came to the lab had 0.00‰). The second assistant prepared the drinks and the randomized assignment to the experimental condition. The third assistant ensured all documents were signed before the study and served the drinks (unaware of the experimental condition).

When participants arrived at the laboratory, they signed their consent, and the first assistant recorded their weight and measured their blood alcohol level using a breathalyzer (AlcoDigital P100). Following this, participants completed a demographic survey that included questions about age, marital status, employment status, religion, political views, subjectively perceived social status, and any diagnoses of coronavirus disease 2019 (COVID-19) for themselves, close family members, and friends.

Next, all participants were instructed to consume the provided drink (see description below for placebo, control, and alcohol groups) within a period of up to 10 min. They were then asked to watch two emotionally neutral movie clips, each lasting 51 min: (a) “The World From Above,” Season 10, Episode 6, titled “Iceland—From Vatnajokull National Park to Gullfoss Waterfall” and (b) “The World From Above,” Season 4, Episode 7, titled “Yellowstone National Park.” The purpose of this was to allow time for alcohol absorption. After the movies, participants' blood alcohol levels were measured again. In the placebo condition, a specially designed broken breathalyzer was used, which emitted the same sound and light signals as the breathalyzer in the experimental group.

Following these procedures, participants completed the main survey, which took approximately 30 min on average. The study concluded with a debriefing session and a final measurement of blood alcohol concentration (BAC). After the debriefing, participants were also asked to guess which condition they had been assigned to. Participants in the experimental condition had to wait until sober or arrange transportation home with a sober driver.

Participants in the control condition consumed a non-alcoholic drink that only contained juice. Participants were told that there was no alcohol in their drinks. Participants in the placebo condition were told that there was alcohol in the drink but also consumed a non-alcoholic drink only containing juice. To create the impression of alcohol consumption, the drink was sprayed with alcohol. Participants in the alcohol condition consumed an alcoholic drink prepared to contain 1.6 g 7of alcohol at 40% strength for each 1 kg of the participant’s body weight. The drink was mixed with the same juice as in other conditions.

## Statistical analysis

### Manipulation check

The effectiveness of the experimental manipulation was investigated by directly asking participants in which condition they thought they were after explaining to them the aim of the study: control (“I didn’t drink alcohol”), placebo (“You told me I was drinking alcohol, but it wasn’t there”) or alcohol condition (“I drank alcohol”). We also controlled for blood alcohol levels by using a breathalyzer before the study, after the movie, and after the survey.

### Differences between the experimental, placebo, and control groups

We planned to submit the responses on the Moral Foundations Sacredness Scale to a 3 (Alcohol Group: alcohol/ no alcohol/ placebo, between-subjects) × 5 (Foundation: Care/ Fairness/ Loyalty/ Authority/ Purity, within-subjects) mixed ANOVA.

## Results

### Manipulation check

We compared experimental groups by breathalyzer data taken after the movie clip but before the main survey. Only the experimental group was intoxicated (*M* = 0.54‰, *S.D.* = 0.12‰), and participants in all other conditions were sober (*M* < 0.001‰). All pairwise comparisons between the experimental groups and other conditions were significant at *p* < 0.001. We also asked participants to self-classify to a condition they believe they were assigned. Participants were correct in guessing that they belonged to the control condition (99.1%) and the experimental condition (96.3%) but far less accurate in the placebo condition (65.8%), *χ*^2^(2) = 65.72, *p* < 0.001. Thus, although our placebo manipulation was not perfect, similar to previous studies using placebo and alcohol conditions (Bodnár et al. 2021; Hróbjartsson and Gøtzsche 2004; Lachenmeier et al. 2016; Mendelson et al. 1984; Sayette et al. 1994; Schlauch et al. 2010), the accuracy in guessing whether alcohol was indeed consumed was the lowest in the placebo condition and drifted toward the guessing accuracy of 50%.

### Differences between the experimental, placebo, and control groups

Descriptive statistics of the Moral Foundations Sacredness Scale for all study groups are presented in Table 1. The supplementary materials show descriptive statistics divided by sex (Table S1).

**Table 1:** Descriptive statistics of Moral Foundations Sacredness Scale for three study groups

Moral Foundation	Control *n* = 109		Placebo *n* = 114		Experimental *n* = 106	
	*M* (*SD*)	Min/Max	*M* (*SD*)	Min/Max	*M* (*SD*)	Min/Max
Care	6.33 (0.76)	3.75/7.00	6.03 (0.98)	2.50/7.00	6.01 (1.04)	2.50/7.00
Fairness	5.73 (1.30)	1.75/7.00	5.66 (1.10)	2.50/7.00	5.86 (1.10)	2.50/7.00
Loyalty	6.05 (0.97)	2.50/7.00	5.93 (0.98)	3.00/7.00	5.96 (1.09)	2.75/7.00
Authority	5.00 (1.40)	2.00/7.00	4.68 (1.40)	1.00/7.00	5.06 (1.39)	1.00/7.00
Purity	5.98 (1.01)	2.00/7.00	5.75 (1.19)	1.00/7.00	5.43 (1.35)	1.00/7.00

Responses on the Moral Foundations Sacredness Scale were submitted to a 3 (Alcohol Group: alcohol/ no alcohol/ placebo, between-subjects) × 5 (Foundation: Care/ Fairness/ Loyalty/ Authority/ Purity, within-subjects) mixed ANOVA. The main effect of the Alcohol Group was not significant, *F*(2, 326) = 1.647, *p* = 0.194, *η*^*2*^_*p*_ = 0.010, but the interaction between Foundation and Alcohol Group was statistically significant, *F*(8, 1304) = 3.940, *p* < 0.001, *η*^*2*^_*p*_ = 0.024. We found differences between groups in the sacralization of care and purity but not in fairness, loyalty, or authority (Table 2).

**Table 2:** Main effects observed in the study

Moral Foundation	Group effect		
	*F*	*p*	*η*^*2*^_*p*_
Care	4.021	0.019	0.024
Fairness	0.804	0.448	0.005
Loyalty	0.379	0.685	0.002
Authority	2.308	0.101	0.014
Purity	5.648	0.004	0.033

Post hoc comparisons with Tukey corrections indicated that participants in the control condition scored higher in care than participants in the experimental condition did (*M*_controls_ = 6.33, 95% LCI = 6.18, UCI = 6.47 vs. *M*_*experimentals*_ = 6.01, 95% LCI = 5.80, UCI = 6.21), *t* = 2.534, *p* = 0.031, *d* = 0.346, and higher than participants in placebo condition (*M*_controls_ = 6.33, 95% LCI = 6.18, UCI = 6.47 vs. *M*_*placebo*_ = 6.03, 95% LCI = 5.85, UCI = 6.21), *t* = 2.373, *p* = 0.048, *d* = 0.318. Moreover, participants in the control condition reported being higher, on average, in purity than participants in the experimental condition did (*M*_controls_ = 5.98, 95% LCI = 5.79, UCI = 6.17 vs. *M*_*experimentals*_ = 5.43, 95% LCI = 5.17, UCI = 5.69),* t* = 3.348, *p* = 0.003, *d* = 0.457. Next, we applied both Bonferroni (Simes 1986) and Benjamini-Hochberg (Benjamini and Hochberg 1995) corrections procedures to see which raw *p* values are still significant. All above *p* values were significant using the Benjamini–Hochberg procedure with *Q* = 0.25 false discovery rate. After employing Bonferroni corrections, only the results for purity were still significant. We want to emphasize that we measured the degree to which the foundation is sacralized. Therefore, our data do not indicate that one participant has a higher level than another in a specific foundation.

Next, we conducted exploratory analyses, excluding participants who correctly guessed that they were in the placebo condition, and we observed the same pattern of results for care *F*(2, 251) = 3.856, *p* = 0.022, *η*^*2*^_*p*_ = 0.030, and for purity *F*(2, 251) = 5.266, *p* = 0.006, *η*^*2*^_*p*_ = 0.040. We also did not find differences in fairness, loyalty, and authority. We discuss the results and limitations of the guessing question below. Lastly, we conducted an exploratory analysis to identify the items most differentiated the participants (Table S2). For the care foundation, it was the item “Kick a dog in the head hard,” and for the purity foundation: “Get a blood transfusion of 1 pint of disease-free, compatible blood from a convicted child molester”.

## Discussion

Alcohol affects how people behave, feel, and think (Heath and Hardy-Vallée 2015), and, in turn, it may affect how they perceive the social world, including what is right or wrong. Because moral judgments are based on emotional and cognitive processes (De Neys and Pennycook 2019), we asked if alcohol may affect how individuals sacralize moral foundations. We hypothesized that alcohol intoxication would make participants sacralize five moral foundations less than sober participants from control and placebo groups. We tested a large sample and found significant differences in the care and purity foundations. Intoxicated participants showed lower sacralization for the care foundation, requiring fewer incentives to be willing to harm others. They also showed lower sacralization for the purity foundation, being more willing to engage in impure behaviors. The effect size for the care foundation was small, while the effect size for the purity foundation was closer to medium. Specifically, intoxicated participants were more willing to declare they would “kick a dog in the head, hard” (Graham and Haidt 2012). Interestingly, alcohol only affected the sacralization of these two moral foundations and did not influence the self-reported willingness to violate fairness, loyalty, and authority. Future studies could shed light on these findings. However, the results we obtained are coherent.

Regarding the sacralization of the care foundation, it has been empirically confirmed that intoxicated individuals exhibit more violent behavior (Pernanen 1991). Additionally, alcohol has been found to put people in a better mood, and a positive mood has been associated with harmful behaviors (e.g., Noval and Stahl 2017; Valdesolo and Desteno 2006).

Concerning the sacralization of the purity foundation, studies have shown that some individuals who consume alcohol exhibit less sexual control (Heath and Hardy-Vallée 2015) and are more prone to committing crimes, including sexual abuse (Martin 2001). The purity foundation is closely related to saint values, as the psychology of disgust and contamination shapes this foundation. It underlies the widespread idea that the body is a temple that can be desecrated by immoral activities and contaminants (Haidt 2001). Therefore, it is likely that we observed that intoxicated participants were more willing to engage in deviant sexual behaviors.

Regarding the other three foundations, we did not observe any effects of alcohol on their sacralization, contrary to our hypothesis. This may be attributed to the fact that these foundations are typically the most important for people, as suggested by previous studies (Atari et al. 2020; Schein and Gray 2018). It is also possible that some moral foundations are more stable than others and may be influenced differently by various factors, including individual differences in values, beliefs, education, culture, etc. Furthermore, since we conducted our experiment on non-addicted participants, we cannot determine how long-term drinking might alter the level of sacralization for the five moral foundations. More studies are needed to investigate how alcohol intoxication impacts morality, including the sacralization of moral foundations.

Additionally, it is worth highlighting that alcohol may have made people less socially inhibited. It is well-known that morality questionnaires are highly correlated with socially desirable responses. Therefore, alcohol might have reduced participants’ readiness to give socially desirable answers.

Lastly, it is essential to note that we tested participants' declarations, so it is difficult to predict whether participants would engage in the declared behaviors. This aligns with the theory of planned behavior and the complex relationship between intentions and behaviors (Ajzen 1985; Kan and Fabrigar 2017). Moreover, a body of evidence demonstrates that individuals with high psychopathic traits possess the capacity to understand right or wrong, yet they still engage in unethical behaviors, including crimes (Aharoni et al. 2014; Cima et al. 2010). However, there is also evidence that higher psychopathic traits are related to specific moral judgments (Paruzel-Czachura and Farny 2023).

Roughly 40% of incarcerated inmates for violent offenses were under the influence of alcohol during committing the crime (Alcohol Rehab 2022). Thus, our results may be helpful for researchers to understand better the immoral behaviors under the influence of alcohol, including violent behaviors and sexual crimes (Exum 2006; Greenfield 1998; Heath and Hardy-Vallée 2015; Pernanen 1991). Past studies have already focused on criminal behaviors under the influence of alcohol, but our study takes a step back by showing that the change also appears in making decisions about right or wrong. And every immoral behavior, including crime, is preceded by such a decision. That is why we need more studies on decision-making under the influence of alcohol to understand the complicated patch from just having one alcoholic drink (which was the case in our study) to committing a crime. If even one alcoholic drink changes how people think about right or wrong, what happens after a few of them? Because of ethical reasons, we cannot conduct this kind of research with very high doses of alcohol, but thanks to our study, we see how strongly alcohol affects such basic moral judgments, like moral foundations, if even one drink impacts those judgments (Haidt 2001).

Our study is not free from limitations. First, we measured moral foundations in only one way, i.e., by asking how much money participants should receive to violate a moral foundation. It would also be worth measuring other nuances about moral foundations, for example, by including differences in emotional and physical care (Clifford et al. 2015) or using a new way of measuring which foundation is more relevant for a person than the other (Jach et al. 2023). Second, not all participants believed they were in a placebo condition. It is pretty hard to convince people that they consumed alcohol because the experience of alcohol intoxication is quite common, and many challenges with using a placebo in alcohol studies have previously been identified (Bodnár et al. 2021). Still, this problem should be considered when interpreting the results. We asked participants to guess the condition after debriefing, so it is also possible that during the study, they believed to be in the alcohol condition, but after the debriefing, they decided to choose the placebo group. We did not control this issue, and future research should consider adding a question about this or some advanced interview. Third, we only tested one dose of alcohol. Although it was higher than in some previous studies (e.g., Francis et al. 2019), future studies should test different doses to understand when exactly alcohol impacts moral judgments. Fourth, we used only one scale, which measures the degree to which people sacralize moral foundations. Past research showed no effects of alcohol on utilitarian moral judgment (Arutyunova et al. 2017; Francis et al. 2019; Paruzel-Czachura et al. 2023a), self-perception including the trait of aggressiveness (Paruzel-Czachura et al. 2022) and self-importance of moral identity (Paruzel-Czachura et al. 2023c), so we need more studies to understand better which types of moral judgments, decisions, or behaviors alcohol may impact. Lastly, future studies could also test how problematic drinking (including addiction) may impact our morality and test other possible factors related to this problem, for example, sleep deprivation (Chaput et al. 2012), delay discounting (Lee and Liao 2022), or appetite and overeating (Kozak and Fought 2011; Scott et al. 2020; Yeomans 2010).

## Conclusions

Perceptions of good and evil help people successfully function in the social world, but sometimes they may guide them to do something immoral. We showed that even one strong alcoholic drink changes the willingness to do harmful and impure behaviors, like kicking the dog in the head. However, alcohol did not change the willingness to stand against authorities or be disloyal or unfair. Because moral judgment precedes immoral behaviors, our results may help explain why some people under the influence of alcohol break the rules by doing things they would never do when sober.

## Supplementary Information

Below is the link to the electronic supplementary material.

Supplementary file1 (DOCX 17 KB)

## Data Availability

Data is available at https://osf.io/bwg6m/?view_only=None.
