# Supplementary material for: Alcohol and morality: one alcoholic drink is enough to make people declare to harm others and behave impurely
Source: Psychopharmacology (Berl). 2023 Aug 9;240(10):2163–72. doi: 10.1007/s00213-023-06438-z (PMC10506948; doi:10.1007/s00213-023-06438-z)
Supplement: Supplementary file 1 — Supplementary file1 (DOCX 17 KB) [file 213_2023_6438_MOESM1_ESM.docx]

**Table S1**

*Descriptive Statistics of Moral Foundations Sacredness Scale for Three Study Groups Divided by Sex*

|  | Control *n* = 109 *M* (*SD*) | | | Placebo *n* = 114 *M* (*SD*) | | Experimental *n* = 106 *M* (*SD*) | |
| --- | --- | --- | --- | --- | --- | --- | --- |
| Moral Foundation | | Men | Women | Men | Women | Men | Women |
| Care | 6.16 (0.72) | | 6.51 (0.77) | 5.68 (1.07) | 6.38 (0.73) | 5.58 (1.20) | 6.42 (0.63) |
| Fairness | 5.52 (1.35) | | 5.94 (1.23) | 5.46 (1.20) | 5.86 (0.96) | 5.57 (1.26) | 6.15 (0.83) |
| Loyalty | 5.88 (1.06) | | 6.22 (0.83) | 5.74 (1.02) | 6.12 (0.90) | 5.75 (1.22) | 6.17 (0.91) |
| Authority | 4.63 (1.44) | | 5.38 (1.27) | 4.33 (1.42) | 5.03 (1.30) | 4.51 (1.40) | 5.60 (1.15) |
| Purity | 5.79 (1.10) | | 6.18 (0.86) | 5.45 (1.32) | 6.05 (0.98) | 5.00 (1.41) | 5.86 (1.15) |

**Table S2**

*ANOVA Analyses for Items of Care and Purity Subscales*

| Items |  |  | *F* |  | *p* |  | *η²_p_* |  |
| --- | --- | --- | --- | --- | --- | --- | --- | --- |
| Care: Kick a dog in the head hard |  |  | 3.705 |  | 0.026 |  | 0.022 |  |
| Care: Shoot and kill an animal that is a member of an endangered species |  |  | 2.665 |  | 0.071 |  | 0.016 |  |
| Care: Make cruel remarks to an overweight person about his or her appearance |  |  | 0.967 |  | 0.381 |  | 0.006 |  |
| Care: Stick a pin into the palm of a child you don’t know |  |  | 3.483 |  | 0.032 |  | 0.021 |  |
| Purity: Sign a piece of paper that says “I hereby sell my soul, after my death, to whoever has this piece of paper” |  |  | 2.894 |  | 0.057 |  | 0.017 |  |
| Purity: Get plastic surgery that adds a 2 inch tail on to the end of your spine (you can remove it in three years) |  |  | 2.397 |  | 0.093 |  | 0.014 |  |
| Purity: Get a blood transfusion of 1 pint of disease-free, compatible blood from a convicted child molester |  |  | 3.616 |  | 0.028 |  | 0.022 |  |
| Purity: Attend a performance art piece in which all participants (including you) have to act like animals for 30 minutes, including crawling around naked and urinating on stage |  |  | \| 1.274 \|  \| \| --- \| --- \| |  | 0.281 |  | 0.008 |  |
